# Supplementary material for: Suspending Effect on Low-Frequency Charge Noise in Graphene Quantum Dot
Source: Sci Rep. 2015 Jan 30;5:8142. doi: 10.1038/srep08142 (PMC4311243; doi:10.1038/srep08142)
Supplement: Supplementary Information — Supporting information [file srep08142-s1.pdf]

Supporting Information for

Suspending Effect on Low-Frequency Charge Noise in Graphene Quantum Dot

Xiang-Xiang Song, [1,2] Hai-Ou Li, [1,2] Jie You, [1,2] Tian-Yi Han, [1,2] Gang Cao, [1,2]
Tao Tu, [1,2] Ming Xiao, [1,2] Guang-Can Guo, [1,2] Hong-Wen Jiang, [3] and Guo-Ping Guo[1,2]

[1] *Key Laboratory of Quantum Information, CAS, University of Science and Technology of China, Hefei, Anhui 230026, China*
[2] *Synergetic Innovation Center of Quantum Information & Quantum Physics, University of Science and Technology of China, Hefei, Anhui 230026, China*
[3] *Department of Physics and Astronomy, University of California at Los Angeles, CA 90095, USA*

1. Suspension of the device

Figure S1 shows a SEM image of a graphene nanoribbon, which is in contact with the substrate. (This device is not used in the experiment.) Figure S2(a) shows a SEM image of a suspended graphene nanoribbon device after transport measurement. Clearly, the nanoribbon is not pulled down to the substrate during the transport measurement, which is different from the situation in Figure S1. Even though a dc voltage of up to 7 V was applied to the back gate, the graphene nanoribbon still remains suspended. Figure S2(b) shows a SEM image of the same device in Figure S2(a) after AFM measurement. The graphene nanoribbon is tapped down, in contact with the substrate, which is similar with Figure S1.

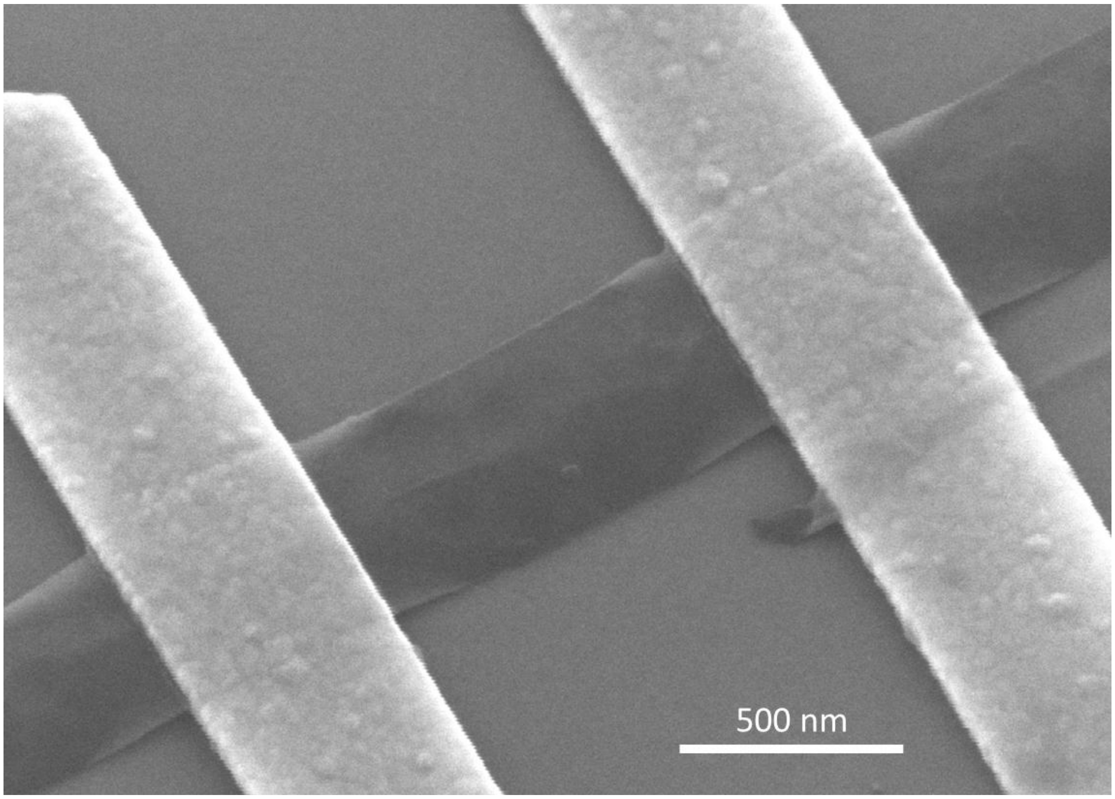

Figure S1. SEM image of a graphene nanoribbon in contact with the substrate surface.

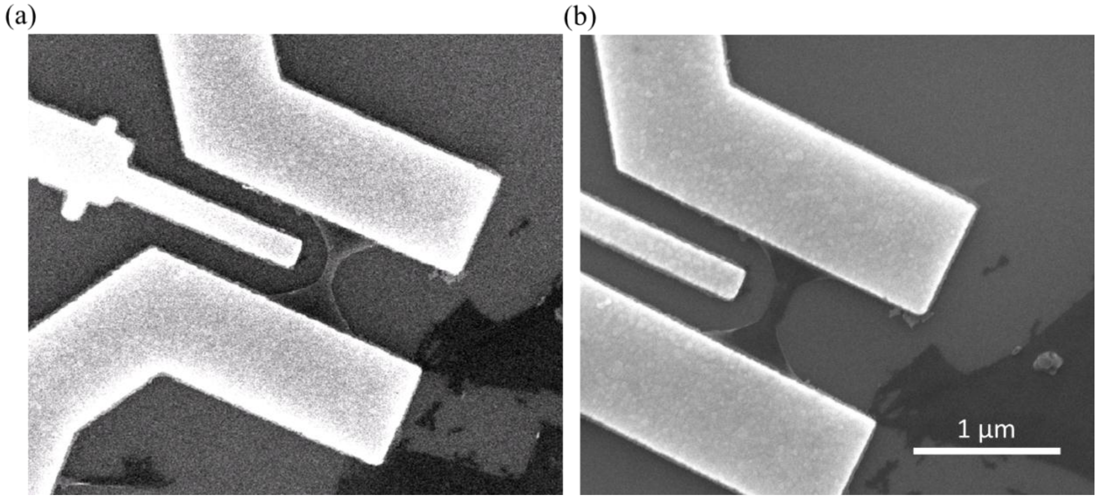

Figure S2. (a) SEM image of a suspended graphene nanoribbon device after transport measurement. (b) SEM image of the same device after AFM measurement.

2. Lever arm

We measure the noise level of five graphene nano-devices, both suspended and regular (unsuspended). As suspend the device above the substrate, the electrical environment is changed, resulting in the difference between the lever arms of the device.

We obtain the lever arm from standard Coulomb diamonds measurement.[S1] As shown in Table S1, we find regular devices have a lever arm almost twice as much as those of suspended devices.

Device Number	Device properties	Lever arm (eV/V)
#1	Suspended	0.07
#2	Suspended	0.08
#3	Regular	0.12
#4	Regular	0.11
#5	Regular	0.12

Table S1. Lever arms of five different nano-devices labeled in Figure 5.

3. Noise spectra analysis using another method

Figure S3 shows two different noise spectra, calculated using the method described in Ref. [S2]. The spectra (red and black) were measured at different regions of the Coulomb

peak, labeled A and B in the inset (similar as in Figure 3). The figure is plotted in log-log scale. All the data points near 50 Hz were removed as they were induced by electricity from the mains. The blue and green dashed lines are the noise spectra obtained from regular and suspended graphene FETs respectively (see Ref. [S2]), showing a difference of an order of magnitude. However, in our experiment, no difference was observed between regular and suspended graphene QDs using this method.

To compare our results to graphene FETs, we focus our attention on spectrum A, since electrons tunnel through the graphene QD at A. Clearly, the noise at A is one (two) order(s) of magnitude larger than the result obtained from regular (suspended) GFETs, respectively.

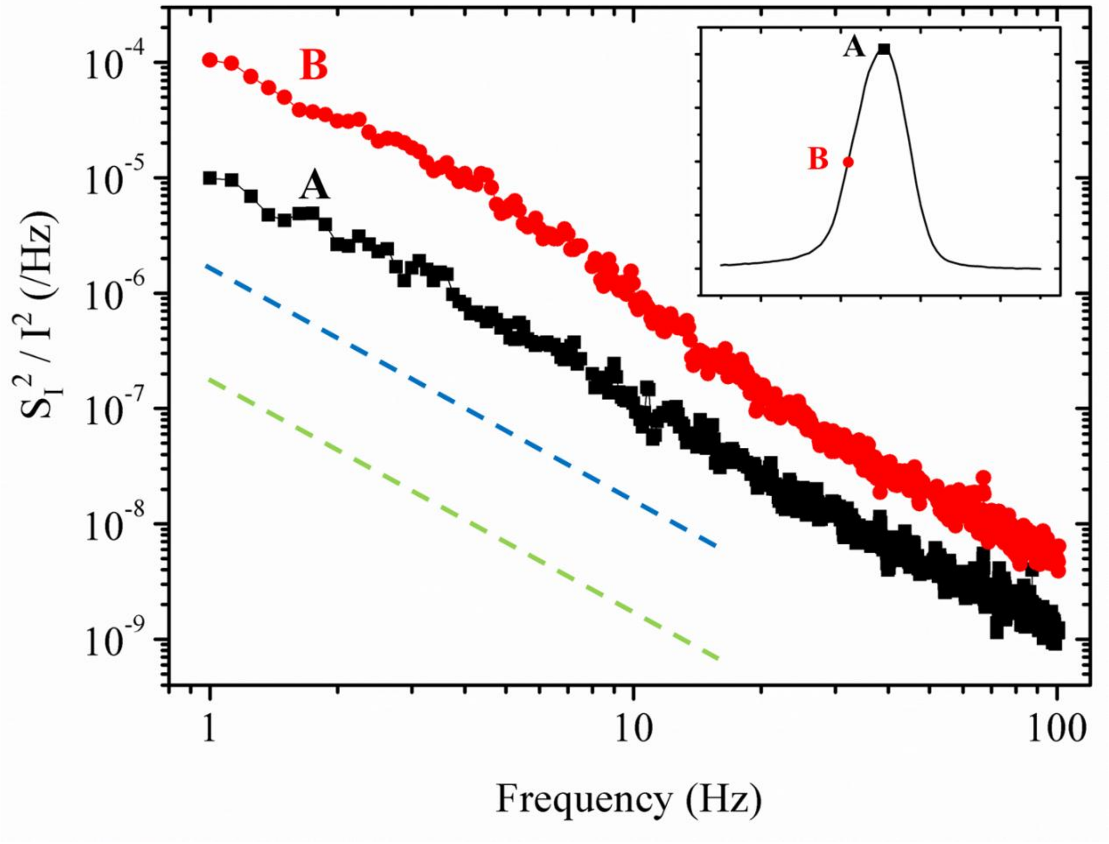

Figure S3. Two different noise spectra measured at different regions of the Coulomb peak, labeled A and B in the inset. The figure is plotted in log-log scale. The blue and green dashed lines are the noise spectra obtained from regular and suspended graphene FETs, respectively (see Ref. [S2]).

We also compared our results to which described in Ref. [S3]. Fitting the spectra with the formula S_I^2/I^2=A/f, we find that the noise power A of our graphene nano-devices (both suspended and unsuspended) is of the order of 10^{-6} to 10^{-5}, which is about one (two)

order(s) of magnitude larger. Furthermore, we obtained our area-scaled noise amplitude (device area (μm^2)$\times$noise power A), as $10^{-2}\times10^{-5}$ (10^{-6})=10^{-7} (10^{-8}). Since we only consider the area of the nanoribbon, the effective area should also include the area of the connection part between the nanoribbon and source-drain contacts, results in larger area-scaled noise amplitude. Compared to the Figure 3 in Ref. [S3], our result is one (two) order(s) of magnitude larger. This result is also consisted with the comparison in the Figure S3, indicating some new sources of noise, such as edge states and surface impurities, influence the performance of the graphene QDs.

REFERENCES:

S1. Schnez, S. *et al*. Observation of excited states in a graphene quantum dot. *Appl. Phys. Lett.* **94**, 012107 (2009).

S2. Cheng, Z. G., Li, Q., Li, Z. J., Zhou, Q. Y. & Fang, Y. Suspended Graphene Sensors with Improved Signal and Reduced Noise. *Nano Lett*. **10**, 1864-1868 (2010).

S3. Zhang, Y., Mendez, E. E. & Du, X. Mobility-dependent low-frequency noise in graphene field-effect transistors. *ACS Nano* **5**, 8124-8130 (2011)
